# Reconstructive Arthroplasty for Malignant Bone Tumors of the Knee—A Single-Center Experience of Functionality and Quality of Life

**DOI:** 10.3390/jcm14176287

**Published:** 2025-09-05

**Authors:** Thilo Khakzad, Michael Putzier, Leonard Thielscher, Nima Taheri, Silvan Wittenberg, Alp Paksoy, Daniel Rau, Sven Märdian

**Affiliations:** Center for Musculoskeletal Surgery, Department of Orthopaedic Surgery, Charité—Universitätsmedizin Berlin, Charitéplatz 1, 10117 Berlin, Germany; michael.putzier@charite.de (M.P.); leonard.thielscher@charite.de (L.T.); nima.taheri@charite.de (N.T.); silvan.wittenberg@charite.de (S.W.); alp.paksoy@charite.de (A.P.); daniel.rau@charite.de (D.R.); sven.maerdian@charite.de (S.M.)

**Keywords:** megaprosthesis, bone tumor, knee, tumor resection

## Abstract

**Background/Objectives:** Resection arthroplasty is well established in treating bone defects following tumor resection, with the distal femur and proximal tibia being its most common localizations. The aim of this study was to analyze the functional outcomes and quality of life following endoprosthetic reconstruction for malignant bone tumors of the knee joint. **Methods:** We retrospectively included all patients treated with an endoprosthetic reconstruction following resection of a malignant bone tumor of the knee at our institution. Functional outcomes (KOOS, OKS, MSTS, and KSS) and health-related quality of life scores [QoL] (SF-36, Karnofsky Index) were evaluated. Chi-square and Fisher’s exact test was used for categorical variables, T-test and Whitney U-Mann tests for continuous variables. Survival was calculated using the Kaplan–Meier curves. **Results:** 32 patients were included. A total of 12 patients had died at the time of follow-up. Among the remaining 20 patients (m:w 17:3), mean follow-up was 8.1 years (range, 8.12 ± 6.8). Mean age at the time of tumor diagnosis was 50 ± 23.3 (10–83) years. According to age, patients were divided into two groups (group C1: <29 years, group C2: >29 years). Group C1 showed significantly better results regarding functional outcome (*p* < 0.05). The anatomic location of the replacement and a revision surgery did not influence the functional outcome (*p* > 0.05). QoL showed no significant differences in subgroup analysis (*p* > 0.05). Primary bone tumors had a significantly better survival (primary tumor: 216.90 months [168.42–265.83]; secondary tumor: 37.03 months [11.71–62.35] *p* = 0.01). Furthermore, pathologic fractures were associated with significantly worse survival (pathologic fracture: 50.24 months [0.00–102.43]; pathologic fracture 190.63 moths [139.28–241.45]; *p* = 0.007). **Conclusions:** Knee resection arthroplasty can offer meaningful long-term functional outcomes and acceptable quality of life in selected patients with musculoskeletal tumors. While the rarity and heterogeneity of such cases remain a challenge, our findings contribute to the growing evidence supporting this complex but limb-sparing surgical option.

## 1. Introduction

The introduction of chemotherapy and the design of interdisciplinary treatment strategies in the 1980s led to a significant increase in survival rates for patients with bone tumors. However, we have seen a significant slowdown in the development of novel therapies in the last decades, so that the usual therapy still consists of the three pillars: radio-therapy, chemotherapy, and surgical intervention [1,2].

The most common form of reconstructive surgical therapy after tumor resection is resection arthroplasty. These prostheses have the advantage of being able to bridge large bone defects and achieve muscular reconstruction via attachment tubes.

The complication rates of large tumor prostheses such as dislocations, infections, periprosthetic fractures, and implant loosening are high, depending on the follow-up period and still represent an unsolved problem to this date [3,4,5,6,7,8,9,10].

Given the rarity of resection arthroplasty in musculoskeletal oncology, studies often include both primary and metastatic bone tumors to achieve sufficient cohort sizes. Despite biological and prognostic differences, this approach reflects clinical practice and has been widely applied in the literature [1,3,4,8,10].

While a number of studies analyzed implant and patient survivorship, as well as complication rates, there is limited knowledge on patients’ functional outcomes and quality of life (QoL). Therefore, the primary aim of this study was to assess long-term functional and patient-reported outcomes, including quality of life (QoL), following tumor-related knee resection arthroplasty. Secondary outcomes included descriptive analyses of complications, pathological fractures, and overall survival. We analyzed functional outcomes and QoL in patients undergoing tumor arthroplasty at a university-based institution.

## 2. Materials and Methods

### 2.1. Ethics Approval

The present study was conducted at a major university clinic with a certified tumor and sarcoma center. Patients with tumor resections at the knee reconstructed using a modular tumor knee arthroplasty between 1996 and 2020 were retrospectively included. The local ethics committee approved the study (EA1/048/22). Furthermore, the study was conducted in accordance with the Declaration of Helsinki.

### 2.2. Patient Characteristics and Follow-Up Examination

All patients at our institution undergo a regular follow-up every 3–6 months since diagnosis. In addition to that, all patients underwent a separate follow-up examination and protocol, exclusively for the purpose of this study. Patients were contacted by phone, mail, or e-mail and invited for an outpatient visit between the years 2020 and 2022. The study protocol included an up-to-date anamnesis, a clinical examination, plain X-rays, measurement of functional outcomes, as well as Patient-Reported Outcome Measures (PROMS) for all patients. Functional outcome parameters and PROMS included Knee Injury and Osteoarthritis Outcome Score (KOOS), Knee Outcome Survey (KOS), Knee Society Score (KSS), Musculoskeletal Tumor Society Score (MSTS), KSS and health-related QoL in the form of Short Form-36 (SF-36) and the Karnofsky Index (KPS). In case patients had died since their last follow-up examination or patients could not be contacted but there was no official report of death, they were considered “lost to follow-up”. Patients who refused to participate were excluded from the study.

Outcome parameters included functional outcomes and QoL, and implant failure according to Henderson et al. [11] as well as survival analysis. In addition, functional outcomes were compared between distal femur and proximal tibia implants, cases requiring revision surgery and those without it, between a younger and an older group based on the median of the follow-up cohort, and among patients with pathological fractures and without.

### 2.3. Subgroup Analysis

Patients were divided into Group A (survival) and Group B (non-survival). Furthermore, we performed subgroup analysis for Group A by dividing the group into young survivors (group C1 < 29 years; C2 > 29 years). Here, the median age of the entire study cohort was used as the cut-off. This data-driven approach was chosen in the absence of an established clinical threshold, allowing for balanced group sizes to facilitate exploratory comparison.

We included patients undergoing knee resection arthroplasty for either primary or metastatic bone tumors. Due to the limited number of metastatic cases in the survival group (3/20), no statistical subgroup analyses between primary and secondary tumor type were performed.

### 2.4. Statistical Analysis

Statistical analysis was performed using SPSS version 25 (SPSS Inc., Chicago, IL, USA). Descriptive statistics were reported as numbers (percentage) or mean (standard deviation). Chi-square and Fisher’s exact test was used for categorical variables, T-test and Whitney U-Mann tests for continuous variables. Survivorship was calculated using the Kaplan–Meier technique. The level of significance was set at *p* < 0.05.

## 3. Results

### 3.1. Patients and Tumor Characteristics

Using the above criteria, 32 patients were eligible for inclusion. Among them, 20 patients (m:w = 10:10) were available for the follow-up protocol. At time of follow-up, 12 patients were deceased. The ASA score of all 32 patients at the time of surgery was 2.3 ± 0.5 (2–3), and the age-adjusted CCI was 5.3 ± 3.3 (2–13). Mean follow-up among all patients was 5.6 years (range, 1.2 to 23.5 years). The mean age at diagnosis was 50 ± 23.3 years.

The follow-up of the survivor group was significantly higher (8.4 years) than that of the deceased group (1.1 years). There was one amputation in each group. No local tumor recurrences occurred. All four cases of systemic recurrence were observed in the non-survivor group. Differences between the survival and non-survival groups are shown in Table 1.

Eleven of the 32 patients included had a secondary bone tumor. Patients in the non-survival group had a significantly higher amount of secondary bone tumors (*p* = 0.008). Eight of the eleven were metastases of renal cell carcinoma, two of bronchial carcinoma, and one of mammary carcinoma. Most of the primary bone tumors were osteosarcomas (10) and chondrosarcomas (4). In addition, there were two cases of Ewing sarcoma and one each of leiomyosarcoma, giant cell tumor, myxofibrosarcoma, rhabdomyosarcoma, and synovial sarcoma.

### 3.2. Primary Outcome Analysis—Functional Outcome and Quality of Life

The overall cohort showed satisfactory results in both functional outcome and quality of life analysis (Table 2).

Statistical analysis of Group A and B showed no significant differences. Group C1 showed significantly better results in terms of functional outcome and quality of life. Except for KOOS, the younger cohort showed better results in all PROMS (KSS, MSTS, and KOS; Table 3 and Table 4). The overall score of SF-36 did not differ significantly in between groups, and subscale analysis revealed significant differences regarding the physical functioning subscale (*p* = 0.007). The KPS also showed a significantly better result for the younger cohort (*p* = 0.011). Anatomical location of the tumor as well as the incidence of revision surgery did not influence the functional outcome.

### 3.3. Secondary Outcome Analysis

#### 3.3.1. Implant-Related Complication

In total, we saw 11 cases of implant-related complications among the 20 patients alive. Eight of these were infections (24.2%) (Type 4 according to Henderson) and three were due to aseptic loosening (9.1%) (Type 2 according to Henderson). There were no dislocations, periprosthetic fractures, or tumor recurrence.

A total of 13 patients in the survival group (n = 20) received a distal femoral implant and in the non-survival group there were 8 (*p* = 0.923). Six survivors underwent proximal tibia reconstruction, in non-survivors this occurred in three cases (*p* = 0.999). In both groups, a total femur arthroplasty was implanted in one case each (*p* = 0.999). Differences in between the groups are further shown in Table 1.

#### 3.3.2. Survival Analysis

**Figure 1:**
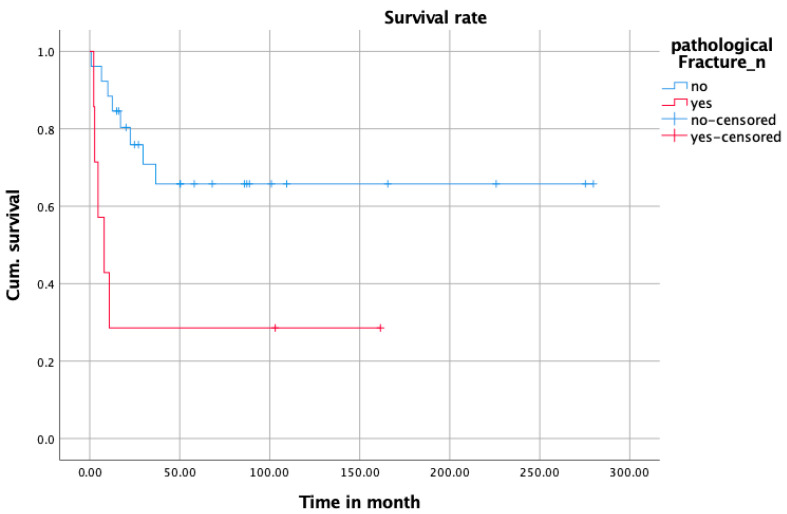
Kaplan–Meier curve of survival between the group with and without pathological fractures.

**Figure 2:**
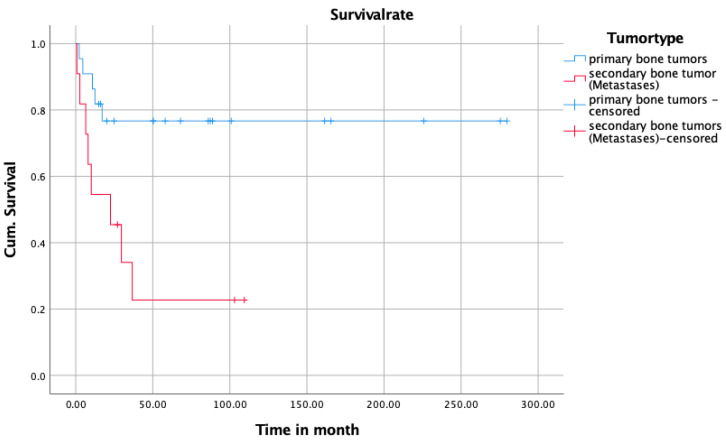
Kaplan–Meier curve of survival between the group with primary and secondary bone tumors.

Primary bone tumors had a significantly better survival (mean survival/month with primary tumor: 216.90 [168.42–265.83]; mean survival/month with secondary tumor: 37.03 [11.71–62.35] *p* = 0.01) (Figure 1). In addition, pathological fractures were associated with a significantly worse survival (mean survival/month with pathological fracture: 50.24 [0.00–102.43]; mean survival/month without pathological fracture: 190.63 [139.28–241.45]; *p* = 0.007) (Figure 2). Test for equality of survival distribution in pathologic fractures was calculated by log-rank test (Mantel–Cox) and showed a significance of 0.010. The significance of the log-rank test for primary and secondary tumors was as high as 0.06.

#### 3.3.3. Example Case

**Figure 3:**
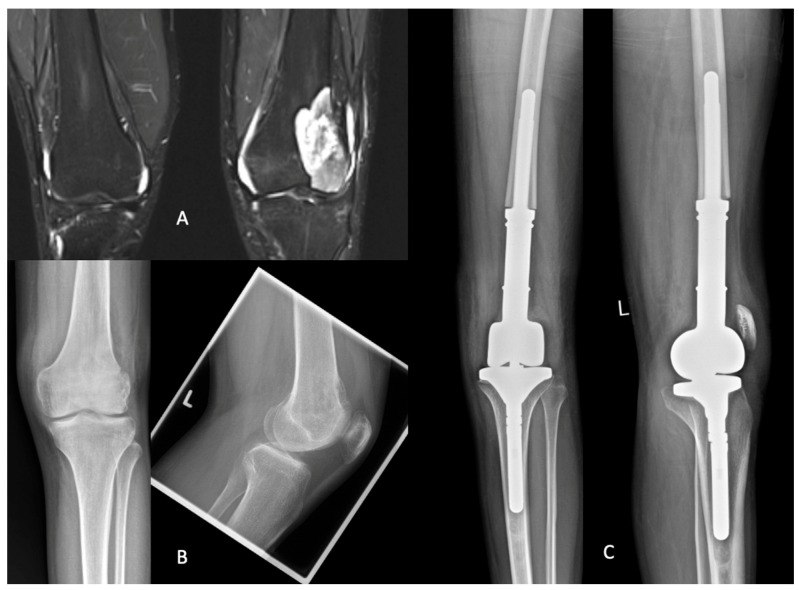
Radiological data of the described patient: (**A**) shows the osteosarcoma in the coronal plane of an MRI; (**B**) shows the osteosarcoma in an X-ray image in two planes; (**C**) shows the postoperative findings two years after the implantation of a tumor prosthesis.

A patient was diagnosed with osteosarcoma of the left distal femur (Figure 3). After an open biopsy confirmed the diagnosis, a multidisciplinary tumor board recommended initiating neoadjuvant chemotherapy according to the EURAMOS protocol. A subsequent PET/CT scan showed no evidence of metastasis. Following several weeks of neoadjuvant chemotherapy, the patient underwent wide extra-articular resection and implantation of a tumor prosthesis. Postoperative care included the continuation of adjuvant chemotherapy and regular monitoring for potential metastases. At the 2.1-year follow-up visit, the patient’s functional scores were recorded as follows: KOOS: 70, OKS: 46, MSTS: 29, KSS-A: 87, KSS: 100, SF-36: 90, and KPS: 100. These results indicate both a good functional outcome and high quality of life.

## 4. Discussion

Most studies describe the success of therapy in terms of implant survival, revision rates, and Patient-Reported Outcome Measures (PROMs), including functional outcomes (e.g., MSTS and OKS) and quality-of-life scores (e.g., SF-36). Furthermore, we evaluated influences on survival rates. For this purpose, we selected patients, which received a tumor endoprosthesis of the knee joint following a resection of a malignant bone tumor until the year 2020. All patients were operated with the same tumor prosthesis type (MUTARS, Implantcast GmbH, Buxtehude, Germany), so that they are comparable. We were able to demonstrate knee tumor arthroplasty as a safe and feasible surgical approach. Younger age is associated with better functional outcomes. Incidences of pathological fractures are associated with worse survival rates.

MSTS scores vary between 75 and 93.4% [5,12,13,14,15]. Wang et al. described an MSTS score of 93.4% in a cohort of patients with osteosarcoma of the knee [12]. In contrast, we demonstrated an MSTS score of 67%. The discrepancy between these results is explained by the shortened follow-up duration in the above-mentioned studies. In addition, reports are often limited to primary bone tumors. They are more likely to occur in young patients, hence better outcomes. In the other studies, the mean age was reported to be between 22 and 30 [12,13,15]. Young patients in our cohort scored a mean value of 80.1% on MSTS. This underlines the influence of age on functional outcome after tumor prostheses. Young patients are generally healthier, recover better, and pursue more active lifestyles, so that muscular regeneration is promoted. To the best of the authors knowledge, this is the first study to report on the association of age at implantation as a prognostic factor for functional outcome.

To quantify the impairment of QoL, patients completed the SF-36 questionnaire. Regarding the total scores in the SF-36, no significant differences are shown between the young and old cohorts in ours. Closer evaluation of the subscales of the SF-36 reveals only a significant advantage regarding physical functioning. The data do not indicate that younger patients have improved QoL after implantation of a tumor endoprosthesis. Contrary to this assumption, young patients show worse scores in emotional functioning and mental health. Psycho-oncological studies describe fear of recurrence (FCR), which is associated with significantly lower psychological scores [16,17]. The rate of implant-related complications in our study was 55%, primarily consisting of infections and aseptic loosening, while no fractures, dislocation, or tumor reoccurrence occurred. In contrast, Wang et al. [12] reported no implant-related complications at the mean follow-up duration of 2 years. A review by Pala et al. reported slightly lower rates of aseptic loosening ranging from 4.9% to 9.6% and a wide range of infections, occurring between 5 and 40% [14]. This high range may represent the variability in patient populations, surgical techniques, follow-up durations, and definitions of complications across different studies. The variability underscores the importance of standardized reporting criteria and more extensive multicenter studies to better understand and mitigate these complications.

Only patients underwent reconstruction with a megaprosthesis, defined here as a modular tumor endoprosthesis, after wide bone resection (e.g., distal femur or proximal tibia), including stemmed fixation and segmental modular components. The subgroup comparison between those with a femoral and those with a tibial component showed no differences. It was suspected that the group with a femoral component might benefit in functionally due to the preservation of the extensor apparatus. However, no functional difference was found here.

Revision surgery after primary knee arthroplasty is associated with functionally worse outcomes [18]. A direct comparison with tumor arthroplasty is not possible in this case because the large resection margins to ensure tumor freedom are associated with large musculoskeletal defects. We could demonstrate that revision surgery is not associated with worse functional outcomes in our collective. This agrees with the results of Lang et al. who demonstrated that revision surgery has no significant impact on physical activity [19]. The discussion of long-term functional outcome after malignant bone tumors has only become possible due to improved interdisciplinary treatment strategies in the last decades. The introduction and use of adjuvant and neoadjuvant chemotherapies in the second third of the 20th century raised the survival rates. According to our results, the survival rate of primary bone tumors is better than that of secondary bone tumors. One reason for this may be that the patients in the cohort with secondary bone tumors also had other metastatic localizations and were in a reduced overall general condition. This conclusion is strengthened as Wang et al. reported only one death in their cohort of 41 patients receiving reconstruction for primary malignant tumors [12]. The better prognosis of primary bone tumors may be attributed to their generally earlier detection and more aggressive localized treatment strategies, compared to secondary bone tumors that often present at a more advanced stage.

Finally, we observed that pathological fractures were more frequent in patients with unfavorable outcomes. Moradi et al. describes similar results in uremic bone tumors, while other studies could not show any correlations [20,21]. We hypothesize that pathologic fracture may be associated with a more invasive and rapid tumor growth and happen at a more progressed stage of the disease. The observed association between pathological fractures and reduced survival must be interpreted cautiously. Although more fractures occurred in the non-survivor group, this trend may reflect confounding by metastatic disease or other unmeasured factors. The prognostic value of pathological fractures remains debated in the literature, and our data do not allow definitive conclusions in this regard.

One of the main limitations of this study is the small sample size, and another is the inclusion of both primary and metastatic bone tumors. While this reflects the clinical reality and aligns with previous high-impact studies in orthopedic oncology, the biological behavior, prognosis, and treatment goals differ substantially between these entities. Moreover, even among primary tumors, there is considerable heterogeneity—for instance between high-grade osteosarcoma, Ewing sarcoma, and low-grade chondrosarcoma—which further complicates outcome interpretation.

The small number of metastatic cases in our cohort, particularly among the long-term survivors, precluded meaningful statistical subgroup analysis. We therefore refrained from drawing causal conclusions based on these data and emphasize the descriptive nature of our findings. Due to the very small number of metastatic cases among long-term survivors (n = 3), a separate subgroup analysis was not feasible or meaningful. The lack of preoperative data and the retrospective study design limit our study. More attention should be paid to the establishment of a multicenter prospective study to give higher-grade evidence based on larger data sets.

## 5. Conclusions

Knee resection arthroplasty can offer meaningful long-term functional outcomes and acceptable quality of life in selected patients with musculoskeletal tumors. While the rarity and heterogeneity of such cases remain a challenge, our findings contribute to the growing evidence supporting this complex but limb-sparing surgical option. Further prospective studies with larger and more homogeneous cohorts are needed to validate these observations.

## Figures and Tables

**Table 1:** Survival vs. non-survival cohort.

	Survival	Non-Survival	*p*
Number	20	12	-
Follow-up in years	8.12 ± 6.8	1.1 ± 0.9	**<0.001**
Age	39 ± 23.2	67 ± 6.9	0.002
Sex male	10	6	0.999
Secondary tumor type/metastasis	3	8	0.008
Pathological fracture	2	5	0.010
Arthroplasty type			
Distal femur	13	8	0.923
Proximal tibia	6	3	0.999
Total	1	1	0.999
Revision surgery			
Infection rate	7	1	0.107
Loosening	3	0	0.270
Cancer recurrence local	0	0	-
Amputations	1	1	0.999
Average surgeries following primary implantation	3.0 ± 3.7	0.6 ± 1.3	0.039
ASA	2.25 ± 0.44	2.3 ± 0.48	0.716
CCI	3.4 ± 2.46	8.4 ± 1.98	**<0.001**

**Table 2:** Functional outcome in group comparison.

	Proximal Tibia (A1)	Distal Femur (A2)	*p*-Value	No-Revision (B1)	Revision (B2)	*p*-Value	Age < 29 (C1)	Age > 29 (C2)	*p*-Value
Number	13	6		9	11		10	10	
KOOS	50.2 ± 17.8	63.4 ± 11.7	0.109	49.5 ± 16.6	60 ± 16.1	0.129	60.9 ± 14.3	44.6 ± 17.3	**0.041**
MSTS	20.0 ± 6.2	20.0 ± 6.2	0.879	20.7 ± 7.0	19.7 ± 5.2	0.565	24.0 ± 3.2	16.4 ± 5.7	**0.004**
OKS	31.4 ± 11.2	35.8 ± 8.12	0.629	31.4 ± 10.7	34.1 ± 9.97	0.620	37.1 ± 9.3	27.3 ± 8.6	**0.032**
KSS A	82.5 ± 11.9	69 ± 15.1	0.819	82.5 ± 11.2	80.5 ± 14.6	0.906	85.6 ± 9.2	77 ± 14.6	0.264
KSS B	76.2 ± 30.7	69 ± 31.7	0.950	75.5 ± 34.7	72.5 ± 26.5	0.661	91.4 ± 14.4	58.1 ± 25.6	**0.010**

**Table 3:** Functional outcome in survival cohort.

Number of Patients (n)	20	Score in %
KOOS ± SD (Range)	54.7 ± 16.7 (25–76)	54.7
OKS	32.7 ± 10.1 (14–46)	68.1
MSTS	20.2 ± 5.9 (6–29)	67.3
KSS A	82.7 ± 11.9 (55–96)	82.7
KSS B	74.1 ± 30.1 (0–100)	74.1
SF-36 overall	63 ± 21 (17–89)	63
KPS	79 ± 18.8 (40–100)	79

**Table 4:** Quality of life.

SF-36	Proximal Tibia	Distal Femur	*p*-Value	No-Revision	Revision	*p*-Value	Age < 29	Age > 29	*p*-Value
SF-36 overall	0.60 ± 0.25	0.70 ± 0.08	0.655	0.64 ± 0.22	0.62 ± 0.21	0.897	68 ± 0.24	58 ± 0.19	0.150
PF	0.52 ± 0.32	0.58 ± 0.29	0.750	0.56 ± 0.36	0.55 ± 0.27	0.839	0.74 ± 0.23	0.37 ± 0.27	**0.007**
PR	0.56 ± 0.48	0.75 ± 0.31	0.470	0.67 ± 0.41	0.55 ± 0.45	0.689	0.68 ± 0.43	0.53 ± 0.45	0.543
BP	0.66 ± 0.32	0.77 ± 0.23	0.524	0.63 ± 0.33	0.74 ± 0.24	0.404	0.79 ± 0.25	0.59 ± 0.31	0.278
GH	0.48 ± 0.28	0.68 ± 0.19	0.217	0.61 ± 0.28	0.52 ± 0.26	0.380	0.61 ± 0.28	0.51 ± 0.27	0.323
SR	0.75 ± 0.27	0.83 ± 0.12	0.883	0.81 ± 0.23	0.77 ± 0.24	0.836	0.85 ± 0.24	0.73 ± 0.23	0.113
EMO	0.74 ± 0.43	0.89 ± 0.27	0.410	0.78 ± 0.37	0.79 ± 0.40	0.928	0.70 ± 0.43	0.87 ± 0.32	0.336
MH	0.46 ± 0.09	0.54 ± 0.08	0.228	0.50 ± 0.125	0.50 ± 0.11	0.999	0.53 ± 0.14	0.48 ± 0.08	0.635
KPS	78.4 ± 20.3	78.3 ± 17.2	0.842	82.2 ± 20.5	76.3 ± 18	0.310	90 ± 9.4	68 ± 19.6	**0.011**

KPS = Karnofsky Performance Status; SF-36 = Short Form Survey 36; PF = Physical Functioning; PR = Physical Role Functioning, BP = Bodily Pain; GH = General Health; VIT = Vitality; SR = Social Role Functioning; ER = Emotional Role Functioning; MH = Mental Health.

## Data Availability

The original contributions presented in this study are included in the article. Further inquiries can be directed to the corresponding author.
